# Prenatal Ethanol Exposure Causes Glucose Intolerance with Increased Hepatic Gluconeogenesis and Histone Deacetylases in Adult Rat Offspring: Reversal by Tauroursodeoxycholic Acid

**DOI:** 10.1371/journal.pone.0059680

**Published:** 2013-03-27

**Authors:** Xing-Hai Yao, Hoa K. Nguyen, B. L. Grégoire Nyomba

**Affiliations:** 1 Department of Internal Medicine, University of Manitoba, Winnipeg, Manitoba, Canada; 2 Department of Physiology, University of Manitoba, Winnipeg, Manitoba, Canada; Bambino Gesu' Children Hospital, Italy

## Abstract

Prenatal ethanol exposure results in increased glucose production in adult rat offspring and this may involve modulation of protein acetylation by cellular stress. We used adult male offspring of dams given ethanol during gestation days 1–7 (early), 8–14 (mid) and 15–21 (late) compared with those from control dams. A group of ethanol offspring was treated with tauroursodeoxycholic acid (TUDCA) for 3 weeks. We determined gluconeogenesis, phosphoenolpyruvate carboxykinase (PEPCK), glucose-6-phosphatase, hepatic free radicals**,** histone deacetylases (HDAC), acetylated foxo1, acetylated PEPCK, and C/EBP homologous protein as a marker of endoplasmic reticulum stress. Prenatal ethanol during either of the 3 weeks of pregnancy increased gluconeogenesis, gluconeogenic genes, oxidative and endoplasmic reticulum stresses, sirtuin-2 and HDAC3, 4, 5, and 7 in adult offspring. Conversely, prenatal ethanol reduced acetylation of foxo1 and PEPCK. Treatment of adult ethanol offspring with TUDCA reversed all these abnormalities. Thus, prenatal exposure of rats to ethanol results in long lasting oxidative and endoplasmic reticulum stresses explaining increased expression of gluconeogenic genes and HDAC proteins which, by deacetylating foxo1 and PEPCK, contribute to increased gluconeogenesis. These anomalies occurred regardless of the time of ethanol exposure during pregnancy, including early embryogenesis. As these anomalies were reversed by treatment of the adult offspring with TUDCA, this compound has therapeutic potentials in the treatment of glucose intolerance associated with prenatal ethanol exposure.

## Introduction

Hostile intrauterine environment is now accepted to play an important role in the pathogenesis of obesity and type 2 diabetes. Although alcohol drinking during pregnancy is a known prenatal insult, studies of the effects of fetal alcohol on glucose homeostasis are currently limited. Prenatal alcohol exposure has been reported to associate with alterations of glucose and lipid homeostasis in humans [1], [2] and animals in association with insulin resistance [3], [4], [5], [6], and recent epidemiologic surveys suggest that parental alcoholism is a predictor of obesity in offspring [7]. In animal studies, this insulin resistance is explained by impairment of insulin signaling through the phosphoinositide 3-kinase pathway due to reduced intrinsic tyrosine kinase [6] or inhibition of Akt and protein kinase Cζ phosphorylation by increased expression of the inhibitors Pten (phosphatase and tensin homologue deleted on chromosome 10) and Trb3 (tribbles 3) [8], [9], [10]. Hepatic gluconeogenesis in these animals is increased in association with increased expression of gluconeogenic genes [9], [11], [12]. Insulin resistance could result from oxidative stress, which has been reported in offspring exposed to prenatal alcohol [13], [14], [15]. Insulin resistance could also result from endoplasmic reticulum (ER) stress, and we have shown in liver of rat offspring prenatally exposed to ethanol an increased expression of several ER markers [10], [16].

The increase in Pten and Trb3 activities after prenatal ethanol was explained, not only by their increased gene expression, but also by their reduced acetylation due to increased expression of the class I histone deacetylase 1 (HDAC1) [10]. HDAC1 and the class III HDACs sirtuin (SIRT) 1–4 play a role in the regulation of gluconeogenesis [17], [18]. Among the sirtuins, SIRT2 acts directly on gluconeogenesis by deacetylating PEPCK, whereas SIRT1 acts indirectly on PEPCK through deacetylation of peroxisome proliferator-activated receptor-coactivator 1 alpha and foxo1, and SIRT3 acts indirectly through glutamate dehydrogenase deacetylation [18]. Oxidative stress is one of the factors regulating the acetylation of non-histone proteins [19], including Pten [20] and foxo proteins [21]. Zhao et al [22] have shown that virtually every enzyme involved in glucose metabolism is regulated by acetylation. Recent research suggests that reactive oxygen species (ROS) from mitochondria can increase liver HDAC activity [23], [24], and that ER stress can also induce an increase of certain HDACs [25], [26]. Tauroursodeoxycholic acid (TUDCA) is a taurine conjugate of ursodeoxycholic acid known to reduce cellular stress through its anti-oxidant and anti-ER stress properties [27], [28], [29], [30], [31]. The purpose of this study was to investigate whether prenatal ethanol exposure alters the expression of class II HDACs and SIRT2 as well as the acetylation status of gluconeogenic proteins in adult rat offspring exposed to ethanol *in utero*, and whether the anti-oxidant and anti-ER stress TUDCA can reverse these anomalies.

## Materials and Methods

### Ethics Statement

All studies were approved by the Committee for Animal Use in Research and Teaching of the University of Manitoba prior to commencement of the studies, in full compliance with the Canadian Council on Animal Care who has certified that the animal care and use program at the University of Manitoba is in accordance with the standards of Good Animal Practice.

### Experimental Design

Animal experiments were performed as described before [9], [10], [14], except that ethanol was given for one gestation week instead of throughout pregnancy. Pregnant Sprague-Dawley rats (Charles River Canada, Saint Constant, QC) were randomly assigned: a) to be gavaged with ethanol during the first, second or third gestation week; b) to be gavaged with water instead of ethanol and be pair-fed the amount of chow consumed by the ethanol dams; c) to be gavaged with water and be given free access to chow. After weaning onto normal chow, subgroups of male offspring from ethanol dams were given tauroursodeoxycholic acid sodium salt (TUDCA, 15 mg/kg) by daily IP injection for 3 weeks as of 13 weeks of age (ethanol-TUDCA), whereas the others were given normal saline. At 16 weeks, male rat offspring from each group, representing individual litters, were fasted for 15 h and the procedures described below were performed.

### Glucose Tolerance Test

Rat offspring were fasted overnight and then submitted to IP glucose tolerance test (IPGTT) by 9∶00 a.m. the next morning. Glucose (30% w/v), 2 g/kg body weight, was IP injected and saphenous vein blood (40 µl) was sequentially collected for the determination of glucose (Ascensia Elite XL, Bayer HealthCare, Toronto, ON, Canada) and insulin (Ultrasensitive Rat Insulin Elisa kit, CrystalChem, Downers Grove, IL). The rats were killed by exsanguination, and liver and plasma were stored, respectively, at −80°C and −20°C until used.

### Pyruvate Challenge Test

Gluconeogenesis was estimated using pyruvate challenge test [10], [12]. Briefly, rats were IP injected with sodium pyruvate (2 g/kg) dissolved in saline, and blood glucose was determined with Ascensia Elite XL (Bayer HealthCare) in saphenous vein blood every 30 min for 2 h.

### Preparation of Liver Tissue Extracts

Liver cytosol was prepared as described [10]. Briefly, liver tissue was homogenized in ice-cold buffer containing 20 mM Tris-HCl, pH 7.4, 250 mM sucrose, 1 mM EDTA, 10 mM sodium fluoride, 2 mM sodium vanadate, 2 mg/ml benzamidine, and a protease inhibitor cocktail (Sigma-Aldrich, Mississauga, ON, Canada). After centrifugation at 3,000 g for 10 min at 4°C, the supernatant was centrifuged at 100,000 g for 90 min and the precipitate was resuspended in ice-cold buffer by shearing through a 22-gauge needle, using at least 10 passes until the precipitate was well resuspended.

Liver nuclear fraction was prepared as described previously [10], [32]. Briefly, liver tissue was homogenized in two volumes of ice-cold TKM (50 mM Tris-HCl buffer, pH 7.5, containing 250 mM sucrose, 25 mM KCl, and 5 mM MgCl2). For nuclear protein acetylation assay, liver was homogenized in RIPA buffer (1×phosphate-buffered saline, 1% Igepal, 0.5% sodium deoxycholate, and 0.1% sodium dodecyl sulfate) containing protease and phosphatase inhibitors and deacetylase inhibitors (100 mM trichostatin A, 10 mM sodium butyrate). The homogenate was filtered through four layers of gauze, and 1.0 ml of filtrate was mixed with 2.0 ml of 2.3 M sucrose in TKM. The mixture was then underlaid by 1.0 ml of 2.3 M sucrose in TKM and, after centrifugation at 124,000 g at 4°C for 30 min, the supernatant was poured off and the nuclear pellet was taken up in TKM buffer.

### Oxidative Stress Determination

Oxidative stress was determined using a commercial kit (OxiSelect in Vitro ROS/RNS Assay kit, Cell Biolabs, San Diego, CA), which measures total free radicals, including ROS and reactive nitrogen species, according to the manufacturer’s instructions. In the presence of ROS, the probe 2′,7′-dichlorofluorescein (DCFH) is oxidized into the highly fluorescent 2′,7′-dichlorodihydrofluorescein (DCF). Liver tissues (50 mg/ml in phosphate buffered saline) were homogenized on ice and spun at 10,000 g for 5 min to remove insoluble particles. Duplicate volumes (50 µl) of samples and DCF standard were added to a 96 well plate followed by 50 µl of a catalyst that helps accelerate the oxidative reaction. After incubation for 5 min at room temperature, 100 µl of DCFH solution were added to samples and the incubation continued for 30 min in the dark. DCF fluorescence was read in a Fluostar Optima fluorescence microplate reader (BMG Labtechnologies, Offenburg, Germany) at 480 nm excitation/530 nm emission. The free radical content (nmol/mg protein) of liver samples was determined in comparison with the DCF standard curve.

### HDAC Activity Assay

HDAC activity was measured colorimetrically using a kit from BioVision (Mountain View, CA) following the manufacturer’s instructions, as described [10]. Briefly, 100 µg of nuclear proteins were added to each well of a 96-well plate. A standard curve was prepared using a deacetylated standard included in the kit, with HeLa nuclear extract and trichostatin A as positive and negative controls, respectively. Absorbance was read in an ELISA plate reader at 405 nm and HDAC activity was expressed as µm/µg protein.

### Western Blotting

Antibodies against GADD 153 or C/EBP homologous protein (CHOP, B-3, sc-7351), HDAC3 (40, sc-16290), HDAC4 (H-92, sc-11418), HDAC5 (G-18, sc-5250), HDAC7 (N-18, sc-11489), HDAC4/5/7 (H-273, sc-11421), forkhead transcription factor (FKHR) or foxo1 (H-130, sc-67140), acetylated-FKHR (D-19, 49437), SIRT2 (A-5, sc-28298), PEPCK (P-16, sc-28477), glucose-6-phosphatase (G6Pase, H-60, sc-25840) and β-actin (I-19, sc-1616), as well as goat anti-rabbit IgG-horseradish peroxidase (HRP, sc-2004), donkey anti-goat IgG-HRP (sc-2020) and donkey anti-mouse IgG-HRP (sc-2314) were purchased from Santa Cruz Biotechnology (Santa Cruz, CA). Antibodies to phospho-HDAC4(Ser632)/HDAC5(Ser498)/HDAC7(Ser486) (#3424) and acetylated-lysine (#9441) were obtained from Cell Signaling Technology (Danvers, MA).

Proteins (50 µg/well) were separated by SDS-PAGE and electroblotted onto nitrocellulose membranes. This protein amount was chosen because it was in the linear range of immunodetection for all proteins tested. The blots were blocked with 5% dry milk and incubated overnight at 4°C with the primary antibody at 1∶500 or 1∶1000 dilution. The blots were washed three times for 10 min each in Tris-buffered saline-Tween and then incubated with goat anti-rabbit, donkey anti-goat or donkey anti-mouse IgG-HRP at 1∶2,000 or 1∶3,000 for 1 h at room temperature, and washed three more times for 10 min. Immune complexes were detected using the ECL chemiluminescent detection kit after exposing the blots to a Kodak X-OMAT AR (XAR-5) film. Protein contents were quantified by densitometry using NIH Image software, and the reading was corrected for that of normal control rats.

### Immunoprecipitation

Aliquots of 500 µg protein diluted to 1.0 mg/ml in the RIPA buffer were incubated overnight at 4°C with anti-HDAC4/5/7 or PEPCK antibodies using constant rotation. Protein A sepharose was then added, and the samples were further incubated for 3 h at 4°C. The immune complexes were washed 3 times with RIPA buffer, and after electrophoresis, they were immunoblotted with anti-HDAC3 or anti-acetylated-lysine antibodies, respectively.

### Real-time PCR

Liver PEPCK and G6Pase mRNAs were determined by real time PCR in isolated liver tissues using reagents from InVitrogen (Carlsbad, CA) and a protocol described before [16], [33]. Total RNA was extracted from 100 mg frozen tissue by the Trizol method, and first-strand cDNAs were synthesized from 1 µg total RNA and serial dilutions of standard RNA using Moloney murine leukemia virus reverse transcriptase and oligo(deoxythymidine) random primers. For each sample, the reverse transcription product (30 ng) was amplified by real-time PCR using primers for rat PEPCK (forward: 5'-GCAGAGCATAAGGGCAAGGT-3', reverse: 5'-CCAAAGAAGGGCCGCATAG-3'), G6Pase (forward: 5'-TCGGGAGGAGGGGGAGTGTTTG-3', reverse: 5'-AGCAGCGTGGTCAGGGAAGCAG-3'), and β-actin (forward: 5'-ACCAGTTCGCCATGGATGAC-3', reverse: 5'-TGCCGGAGCCGTTGTC-3') in a final volume of 25 µl containing 12.5 µl SYBR Green PCR master mix. For each standard dilution, 1 µl of cDNA reverse transcript was amplified using primer 1 and primer 2. The Applied Biosystem 7500 thermocycler was used to perform the PCR, with the following cycles: denaturation at 95°C for 10 min followed by 40 cycles of 15 sec at 95°C, 1 min 30 sec at 60°C. Data were analyzed by the ΔΔCt method using ABI 7500 system software, and mRNA levels were normalized to β-actin mRNA.

### Statistical Analysis

Statistical analyses were performed with SPSS software. Data from rat offspring were compared by one-way ANOVA between maternal gestational treatment weeks and treatment groups (normal control, pair-fed, ethanol, and, ethanol-TUDCA) using one-way ANOVA with post hoc Tukey’s honestly significant difference test. Interaction between treatment and week of exposure was determined by two-factor ANOVA to further verify the influence of time of exposure on the effects of ethanol, and is reported in the text only when statistically significant. Insulin concentrations were log-transformed before the analyses. Data are expressed as the mean ± SE. P<0.05 was considered significant.

## Results

### Body Weight

**Figure 1:**
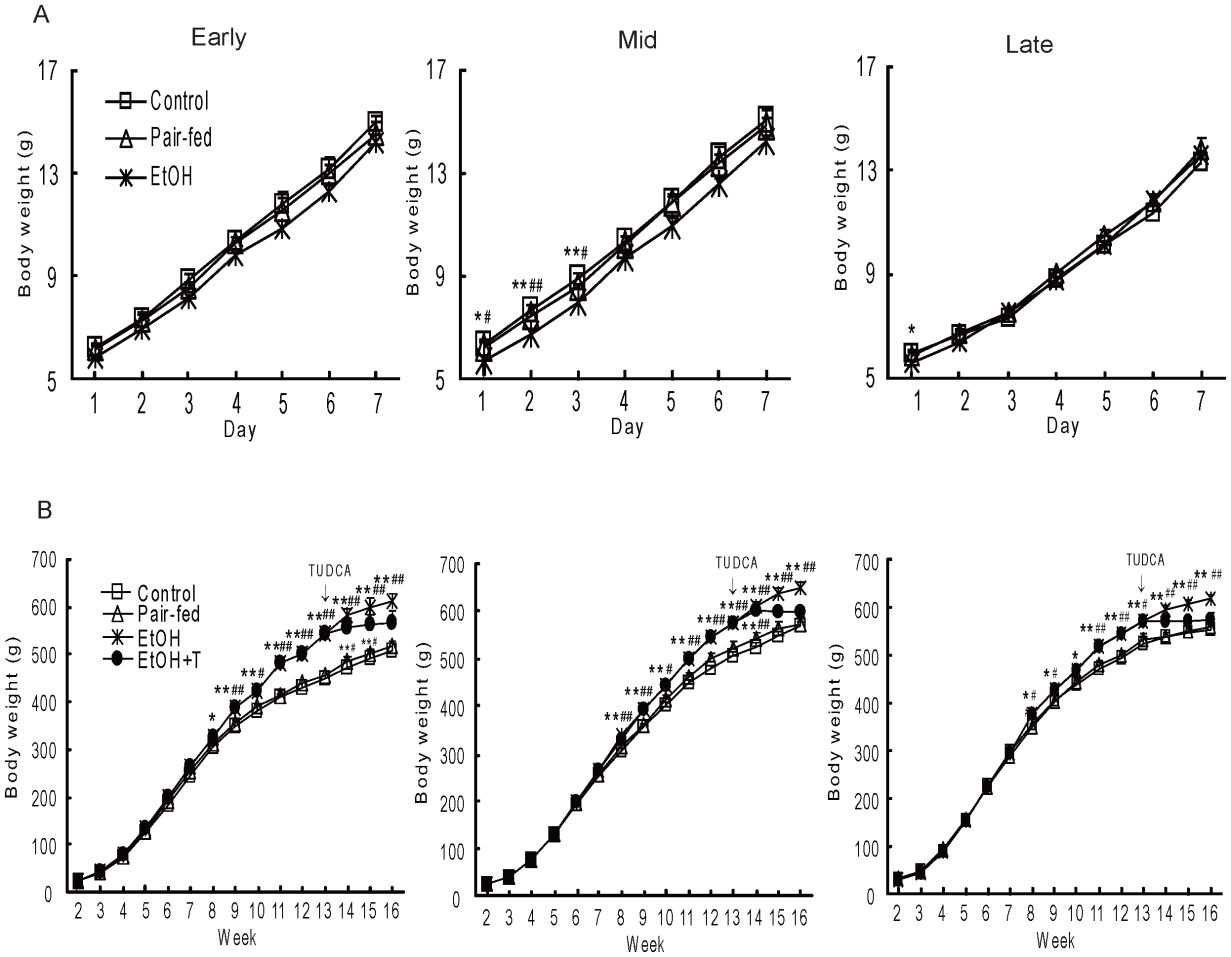
Body weight of male rat offspring during week 1 (A) and weeks 2–16 (B) of life. The offspring were exposed to ethanol (EtOH) or water only during windows of exposure representing early, mid, or late pregnancy. Control and pair-fed offspring were from dams with free access to chow and dams fed the amount of chow consumed by EtOH dams, respectively. TUDCA (T) was given to a subgroup of EtOH offspring for 3 weeks prior to sacrifice. Data shown as the mean±SE, n = 6. *P<0.05, **P<0.01 ethanol (EtOH) *vs.* control; ^#^P<0.05, ^##^P<0.01 EtOH *vs.* pair-fed.

On day 1 of life, offspring from ethanol dams were smaller than those from pair-fed and control dams (P<0.05–0.01) after exposure at early and late gestation (Fig. 1). The weight of ethanol offspring exposed at mid gestation remained lowest onto the third day of life. However, all offspring from ethanol dams progressively gained weight and were heavier (P<0.05–0.01) than the other two groups as of the eighth week of life and this weight gain was offset by 3 weeks of TUDCA treatment (Fig. 1).

### Glucose Tolerance

**Figure 2:**
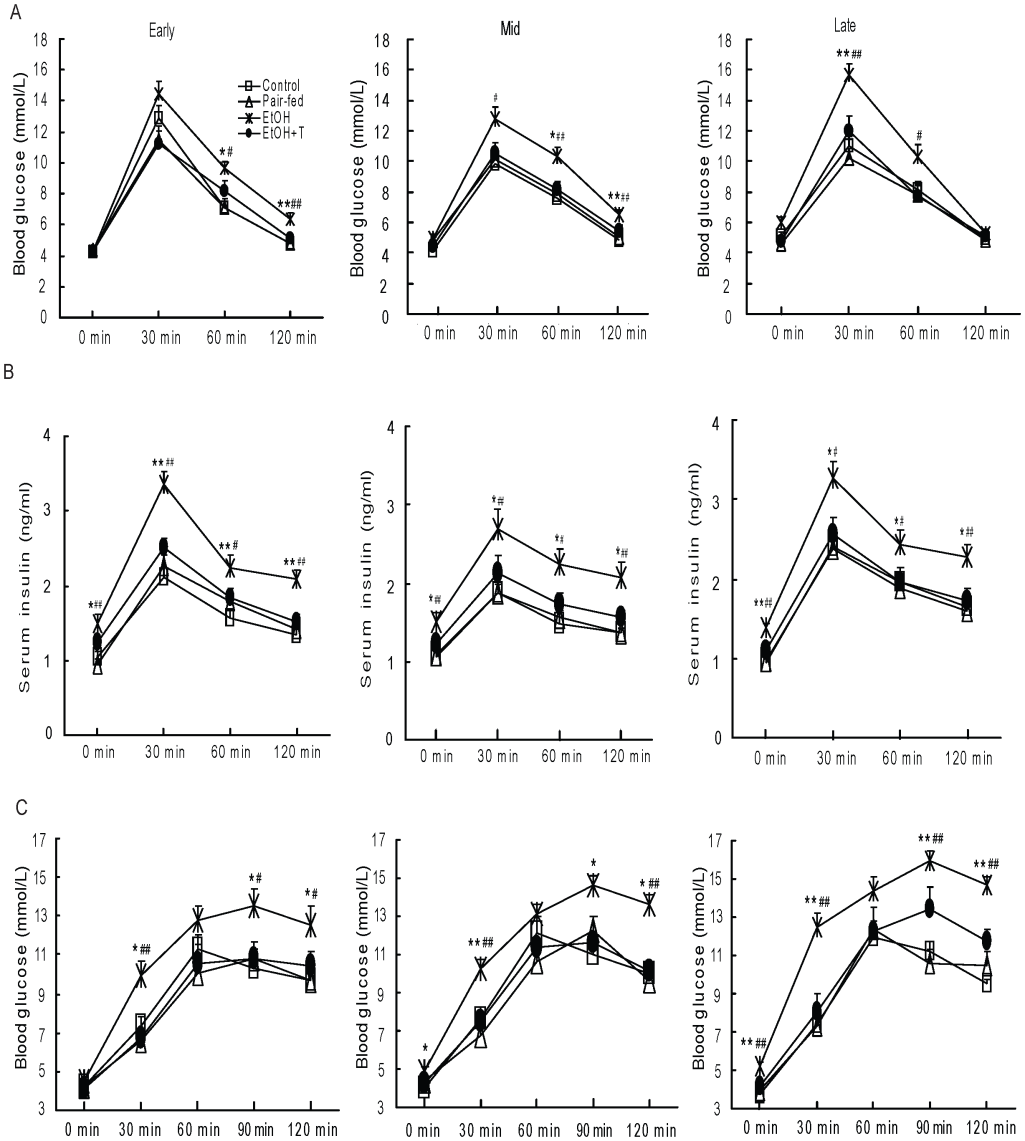
Blood glucose and insulin levels after intraperitoneal injection of glucose (A, B) and blood glucose after intraperitoneal injection of sodium pyruvate (C) in 4 month-old male rat offspring exposed to ethanol (EtOH) or water only during early, mid, or late pregnancy. Control and pair-fed offspring were from dams with free access to chow and dams fed the amount of chow consumed by EtOH dams, respectively. A subgroup of EtOH rats was given TUDCA (T) for 3 weeks before the measurements. Data shown as the mean±SE, n = 6. *P<0.05, **P<0.01 EtOH *vs.* control; ^#^P<0.05, ^##^P<0.01 EtOH *vs.* pair-fed.

After exposure during early pregnancy, serum glucose concentrations were higher (P<0.05–0.01) in ethanol rat offspring than in control and pair-fed rats at 60 min and 120 min of IPGTT (Fig. 2). These glucose differences appeared as of 30 min after exposure in mid pregnancy and were already apparent in the fasting state following exposure in late pregnancy. However, insulin levels during IPGTT were higher (P<0.05–0.01) in ethanol exposed rats than in control and pair-fed rats treated at the same pregnancy stage at all the time points examined. TUDCA treated ethanol rat offspring had similar glucose and insulin levels to those of control and pair-fed groups. Similarly, area under the curves for glucose and insulin were greater (P<0.05–0.001) in ethanol rats than in control and pair-fed groups (Table 1). Comparison of weeks of exposure using two-factor ANOVA showed a time effect on glucose concentrations with a significant interaction (P<0.05) between weeks of exposure and treatment, so that glucoses were highest (slightly) after exposure during the third gestational week. There was no interaction between time of exposure and treatment on insulin concentrations.

**Table 1:** Areas under the curve during IPGTT.

	Control	Pair-fed	EtOH	EtOH+T
**Glucose curve (mmol.min/l)**				
Early	914±40	872±45	1121±54*^##^	923±47
Mid	839±54	870±28	1078±42**^###^	896±42
Late	923±40	867±51	1180±62**^##^	932±72
**Insulin curve (ng.min/ml)**				
Early	190±9	205±6	285±17***^###^	222±9
Mid	161±12	164±8	240±21*^##^	185±16
Late	223±12	218±15	296±21*^#^	233±21

Four month-old male rat offspring were exposed to ethanol (EtOH) or water only during early, mid, or late pregnancy. Control and pair-fed offspring were from dams with free access to chow and dams fed the amount of chow consumed by EtOH dams, respectively. A subgroup of EtOH rats was given TUDCA (T) 3 weeks before the experiments. Data are the mean±SE, n = 6. *P<0.05, **P<0.01, ***P<0.001 EtOH *vs.* control; ^#^P<0.05, ^##^P<0.01, ^###^P<0.001 EtOH *vs.* pair-fed.

### Gluconeogenesis

The effects of prenatal exposure to ethanol during early, mid, and late pregnancy on gluconeogenesis were investigated by measuring blood glucose after pyruvate bolus administration (Fig. 2). Blood glucose following pyruvate administration was higher (P<0.05–0.01) in ethanol rats *vs.* control and pair-fed rats regardless of exposure time, and TUDCA treatment prevented the excessive increase in glucose levels.

### Gluconeogenic Enzymes

**Figure 3:**
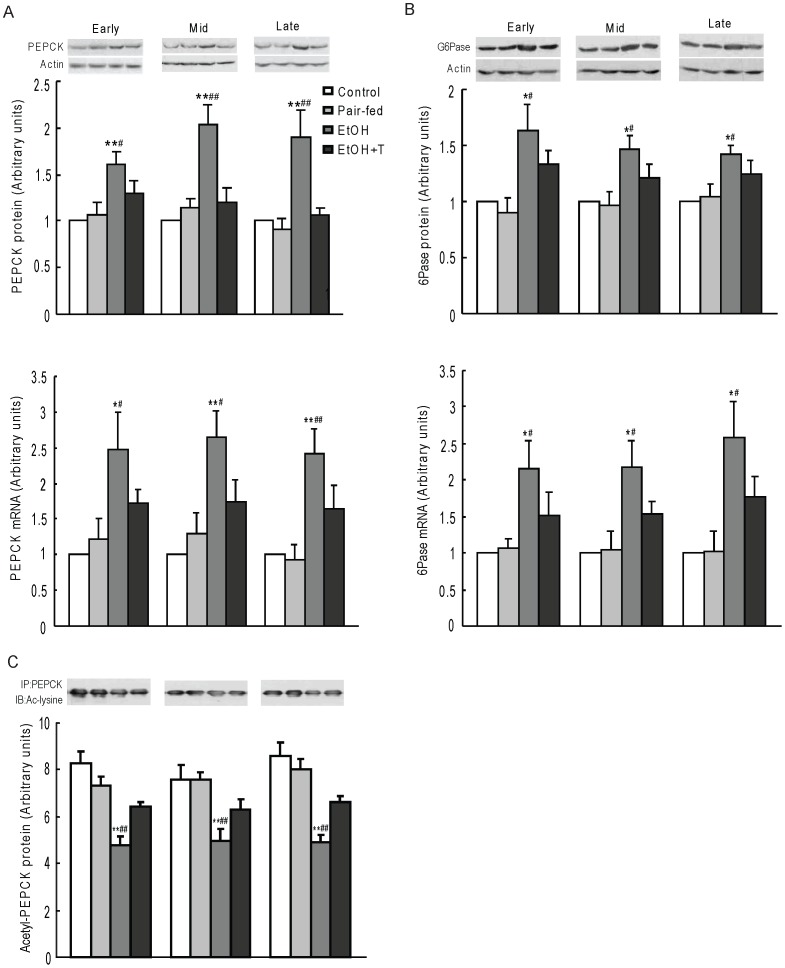
Hepatic nuclear PEPCK (A) and G6Pase (B) expression, and acetylated PEPCK (C) in 4 month-old male rat offspring exposed to ethanol (EtOH) or water only during early, mid, or late pregnancy. Control and pair-fed offspring were from dams with free access to chow and dams fed the amount of chow consumed by EtOH dams, respectively. A subgroup of EtOH rats was given TUDCA (T) 3 weeks before the experiments. Data shown as the mean±SE, n = 6. *P<0.05, **P<0.01 EtOH *vs.* control; ^#^P<0.05, ^##^P<0.01 EtOH *vs.* pair-fed.

PEPCK and G6Pase protein and mRNA levels were higher (P<0.05–0.01) in ethanol rat offspring than in control and pair-fed rat offspring from similar pregnancy stages of exposure, and these protein and mRNA levels were reversed by treatment with TUDCA (Fig. 3). In contrast, acetyl-PEPCK determined by immunoprecipitation with anti-PEPCK antibody followed by probing with anti-acetylated-lysine antibody was decreased (P<0.01) in ethanol exposed rats and increased by TUDCA treatment (Fig. 3).

### Gluconeogenic Histone Deacetylases

**Figure 4:**
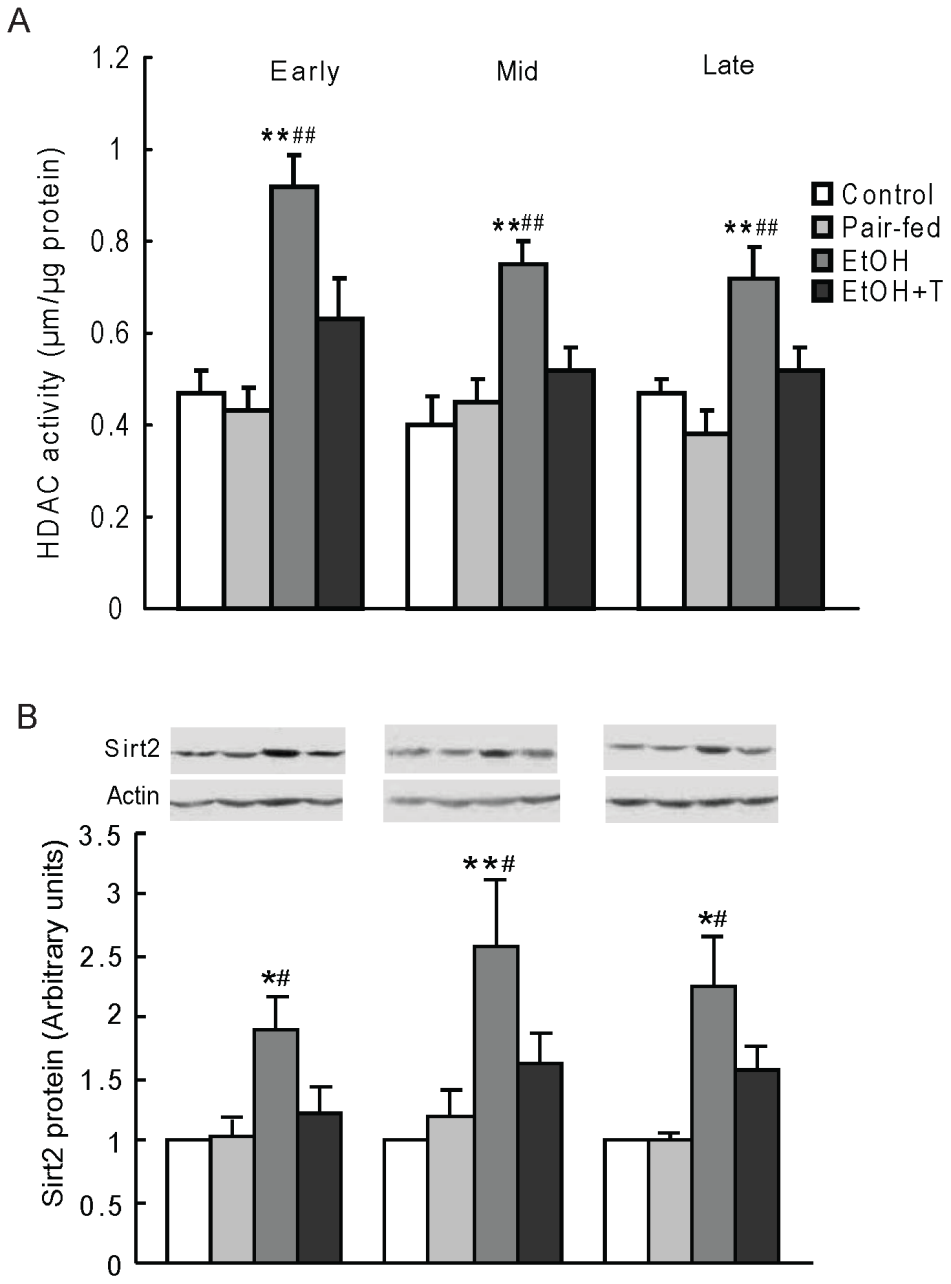
Hepatic HDAC activity (A) and nuclear SIRT2 protein level (B) in 4 month-old male rat offspring exposed to ethanol (EtOH) or water only during early, mid, or late pregnancy. Control and pair-fed offspring were from dams with free access to chow and dams fed the amount of chow consumed by EtOH dams, respectively. A subgroup of EtOH rats was given TUDCA (T) 3 weeks before the experiments. Data are the mean±SE, n = 6. *P<0.05, **P<0.01 EtOH *vs.* control; ^#^P<0.05, ^##^P<0.01 EtOH *vs.* pair-fed.

**Figure 5:**
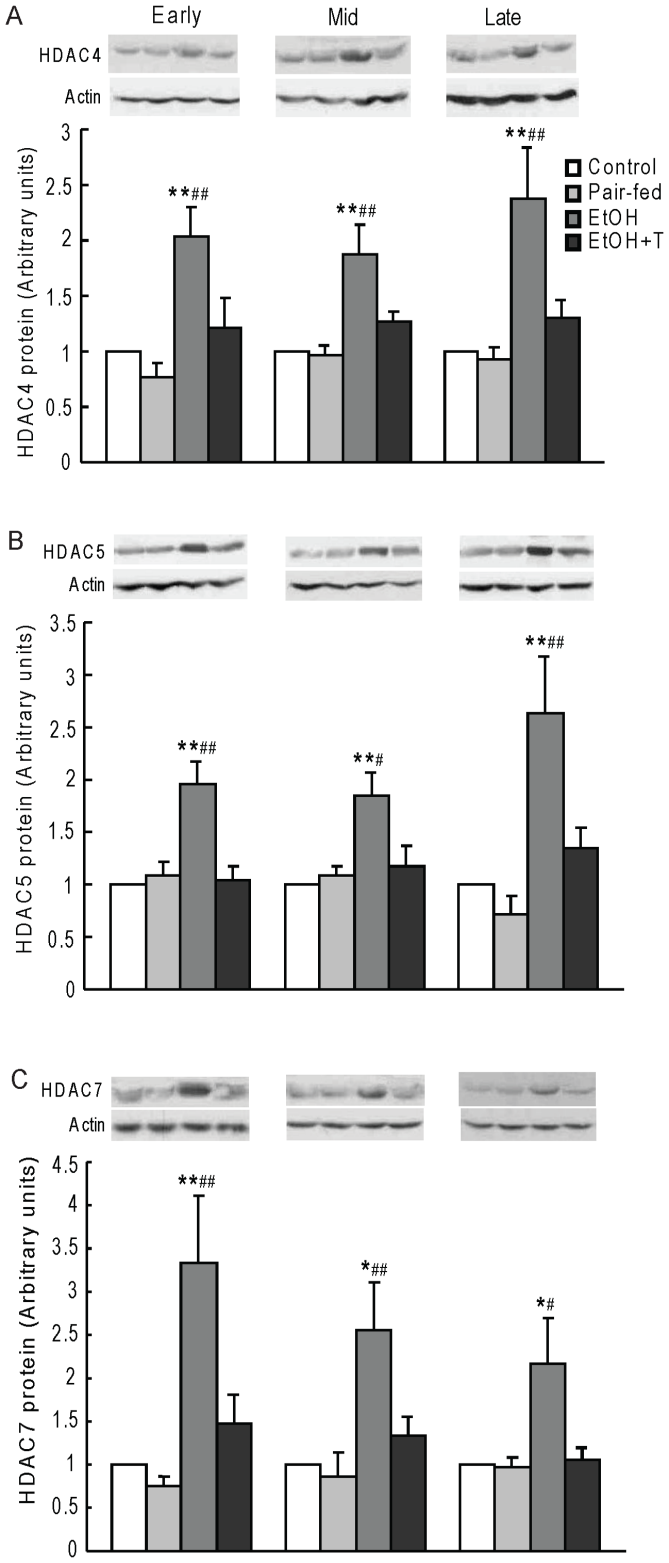
Hepatic nuclear HDAC4 (A), HDAC5 (B), and HDAC7 (C) protein levels in 4 month-old male rat offspring exposed to ethanol (EtOH) or water only during early, mid, or late pregnancy. Control and pair-fed offspring were from dams with free access to chow and dams fed the amount of chow consumed by EtOH dams, respectively. A subgroup of EtOH rats was given TUDCA (T) 3 weeks before the experiments. Data shown as the mean±SE, n = 6. *P<0.05, **P<0.01 EtOH *vs.* control; ^#^P<0.05, ^##^P<0.01 EtOH *vs.* pair-fed.

**Figure 6:**
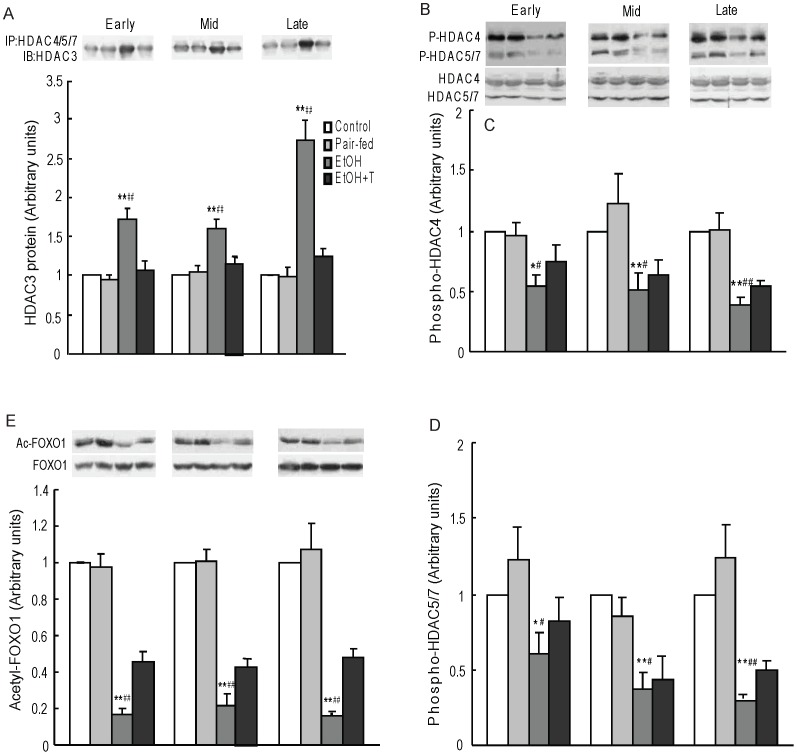
Hepatic nuclear HDAC3 protein content immunoprecipitated (IP) with HDAC4/5/7 antibody (A), representative western blot of cytosolic phospho-HDAC4,5, and 7 (B), cytosolic level of phospho-HDAC4 (C) and phospho-HDAC5/7 (D), and nuclear content of acetyl-foxo1 protein (E) in 4 month-old male rat offspring exposed to ethanol (EtOH) or water only during early, mid, or late pregnancy. Control and pair-fed offspring were from dams with free access to chow and dams fed the amount of chow consumed by EtOH dams, respectively. A subgroup of EtOH rats was given TUDCA (T) 3 weeks before the experiments. Data shown as the mean±SE, n = 6. *P<0.05, **P<0.01 EtOH *vs.* control; ^#^P<0.05, ^##^P<0.01 EtOH *vs*. pair-fed.

PEPCK acetylation status is regulated by the balance between the acetyltransferase p300 and the class III HDACs, sirtuins. We have previously shown a decrease of histone acetyltransferase activity and an increase of HDAC activity with increased HDAC1 expression in the liver of rats prenatally exposed to ethanol [10]. Here, we first confirmed an increase of HDAC activity in ethanol rats *vs.* control and pair-fed rats (P<0.01) regardless of time of exposure during pregnancy (Fig. 4). TUDCA treatment decreased HDAC activity similar to normal controls regardless of the timing of ethanol exposure during pregnancy. We then determined the level of hepatic nuclear SIRT2 protein, which is known to deacetylate PEPCK, and found it to be increased in ethanol *vs.* control and pair-fed rat offspring (P<0.05–0.01), and normalized by TUDCA treatment (Fig. 4). We next determined class II HDAC protein content in nuclear extracts (Fig. 5). The protein levels of HDAC4, 5, and 7 were higher in ethanol rat offspring than in offspring from control and pair-fed dams at equivalent times of exposure during pregnancy (P<0.05–0.01), and TUDCA treatment reversed these increases. Because HDAC4, 5, and 7 are catalytically inactive and are activated when complexed with HDAC3 [34], we tested the interaction of HDAC4,5, and 7 with HDAC3 by immunoprecipitating HDAC4, 5, 7 together and probing for the presence of HDAC3 in the immunoprecipitate (Fig. 6). HDAC3 protein content in this precipitate was significantly greater in ethanol rats than in offspring from control and pair-fed dams (P<0.01), and TUDCA treatment reversed this change. By two-factor ANOVA, there was a significant effect of time on HDAC3 association with HDAC4/5/7 with a strong interaction between week of exposure and treatment (P<0.0001), so that HDAC3 level in this complex was highest after late gestation exposure.

Because HDAC activity can be regulated by phosphorylation in the cytosol, we determined hepatic cytosol HDAC4, 5, and 7 protein phosphorylation state (Fig. 6). Due to the close molecular weights of HDAC5 and 7, these proteins did not resolve into 2 discrete bands. Nevertheless, the results suggest that ethanol rat offspring had decreased phosphorylation of HDAC4, 5, and 7 vs. control and pair-fed offspring from all three stages of prenatal exposure (P<0.05–0.01). However, the reduced phosphorylation of these HDACs was only partially reversed by TUDCA treatment.

### Foxo1 Acetylation

To further investigate the effects of HDAC4/5/7/3 complex on foxo1 acetylation, we measured acetyl-foxo1 protein level by western blotting using acetylated-FKHR antibody (Fig. 6). Acetyl-foxo1 protein level was significantly decreased in ethanol rats than in control and pair-fed rats regardless of time of exposure (P<0.01), and TUDCA partially reversed this change.

### Cellular Stress

**Figure 7:**
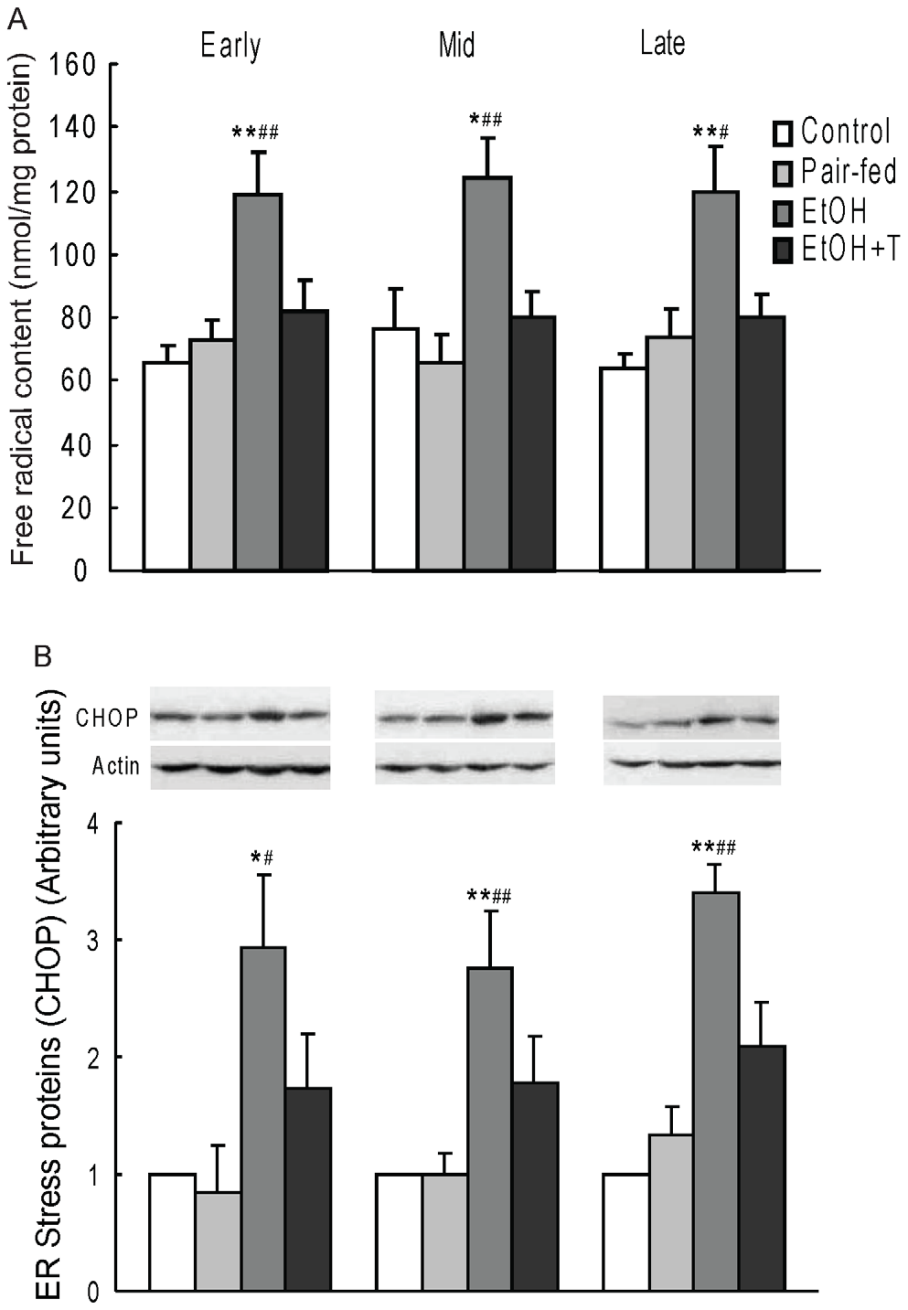
Hepatic cytosol free radical level (A) and ER stress protein (CHOP) level (B) in 4 month-old male rat offspring exposed to ethanol (EtOH) or water only during early, mid, or late pregnancy. Control and pair-fed offspring were from dams with free access to chow and dams fed the amount of chow consumed by EtOH dams, respectively. A subgroup of EtOH rats was given TUDCA (T) 3 weeks before the experiments. Data are the mean±SE, n = 6. *P<0.05, **P<0.01 EtOH *vs.* control; ^#^P<0.05, ^##^P<0.01 EtOH *vs.* pair-fed.

As ethanol may cause oxidative and ER stresses, we measured free radicals and CHOP in rat offspring liver (Fig. 7). We found that prenatal ethanol increased free radical accumulation by 60–90% vs. control and pair-fed rats regardless of the time of exposure (P<0.05–0.01). As with gluconeogenesis, treatment with TUDCA reversed the increase in free radical levels in ethanol rats. We also found that ER stress protein CHOP expression was increased by 155–240% vs. control and pair-fed offspring (P<0.05–0.01) regardless of the time of exposure, and that TUDCA reversed this increase (Fig. 7).

## Discussion

This is the first report that increased gluconeogenesis and glucose intolerance develop in adult offspring after exposure to ethanol during either of the 3 weeks of rat gestation, despite increased insulin levels indicative of insulin resistance. Increased gluconeogenesis was explained by increased expression of major genes PEPCK and G6Pase in the liver, consistent with our previous studies where rats were prenatally exposed to ethanol throughout the whole duration of pregnancy [10], [12]. The current study further shows that adult rats prenatally exposed to ethanol for only one week have increased expression of histone deacetylases involved in gluconeogenesis. In a previous report, adult rat offspring exposed to ethanol throughout pregnancy had increased expression of HDAC1, resulting in reduced acetylation and enhanced activity of Akt inhibitors Pten and Trb3 [10]. Other HDACs have been reported to be increased after ethanol exposure in cell culture systems [24], [35].

Prenatal ethanol could increase HDAC activity by inducing oxidative and ER stresses [13], [14], [36], as shown by the increase in their respective markers, free radicals and CHOP. Other ER markers previously found in this rat model include cytochrome P450 2E1, glucose regulated protein 58, glucose regulated protein 94, hexose-6-phosphate dehydrogenase, and Trb3 [10], [11], [16]. ER stress increases expression of hepatic gluconeogenic enzymes through HDAC dependent mechanisms [37] and some HDACs are known to be upregulated in response to ER stress [25], [26]. Oxidative stress also increases HDAC activity by promoting dephosphorylation of HDAC4, 5 and 7 with as a consequence their translocation into the nucleus where they form a complex that recruits HDAC3, leading to the induction of gluconeogenic genes [23], [24], [38]. HDAC4, 5 and 7 have also been reported to promote the transcription of gluconeogenic genes via deacetylation and activation of foxo family of transcription factors [38]. Similar to the absence of insulin, oxidative stress also results in dephosphorylation of foxo1, leading to its nuclear accumulation and increased transcription of gluconeogenic genes [39]. Recent studies also show that nuclear foxo1 deacetylation by HDAC3 results in increased expression of foxo1 target genes such as PEPCK and G6Pase [38]. The class III HDAC SIRT2 also deacetylates foxo1 and peroxisome proliferator-activated receptor-γ coactivator-1α, two transcriptional inducers of gluconeogenic enzyme gene expression [40], [41], [42]. In addition, SIRT2 activates PEPCK through deacetylation [43], and PEPCK acetylation decreases its stability and inactivates its catalytic activity [22]. We found that prenatal ethanol exposure increases SIRT2 protein expression and PEPCK deacetylation and we confirm our previous reports that prenatal ethanol increases PEPCK expression. We found, in addition, that nuclear HDAC4, 5, and 7 proteins were increased and co-immunoprecipitated with HDAC3 in rats prenatally exposed to ethanol, whereas acetylated PEPCK and foxo1 protein levels were lower. Thus, protein deacetylation plays a central role in increased gluconeogenesis of rats prenatally exposed to ethanol. We propose that prenatal ethanol exposure increases HDAC activity as well as foxo1 and PEPCK deacetylation through an increase of oxidative and ER stresses, resulting in increased gluconeogenesis.

Importantly, the anomalies associated with prenatal ethanol exposure were reversed by TUDCA, a taurine conjugate of ursodeoxycholic acid. TUDCA likely caused these beneficial effects by reducing cellular stress, as it is known to have anti-oxidant properties through stabilization of mitochondrial membranes and function [27], [28] and to have chemical chaperone inhibitory activity on ER stress [29], [30], [31]. Because of the duality of action of this product, it is not possible to determine which one of the ER and oxidative stresses is the primary site for the reduction of gluconeogenesis by this agent after prenatal ethanol exposure. Furthermore, there is an interrelationship between ER and oxidative stresses whereby chronic ER stress is a cause of oxidative stress and, vice-versa, oxidative stress is a cause of ER stress [44], [45]. During ER stress, thiol oxidoreductase enzymes responsible for disulfide bond formation of glycoproteins are upregulated in the ER lumen and catalyze oxidation/reduction reactions, with molecular oxygen as the electron recipient [46].

Another interesting finding in this study is the alteration of glucose regulation regardless of time of prenatal ethanol exposure with, however, a significantly greater effect in offspring exposed during the third gestational week. Interestingly, but by an unknown mechanism, these rats showed a greater association of HDAC3 with HDAC4/5/7 than offspring exposed to ethanol during the first two weeks. These observations are in agreement with the concept that there is no clear-cut fetal window of vulnerability to endocrine dysfunction, although the third and possibly the second trimesters of pregnancy may be more vulnerable [47]. It also has been suggested that the epigenome is vulnerable to ethanol during early embryogenesis, a time when the DNA synthetic rate is high and there is genome wide epigenetic reprogramming [48].

In summary, ethanol exposure for only one week during gestation is followed by long-lasting deleterious effects on cellular stress and glucose regulation in adult rat offspring in association with increased class II HDAC proteins and SIRT2. These anomalies occurred regardless of the time of ethanol exposure during pregnancy, including early embryogenesis, a time when the majority of women are unaware of their pregnancy and therefore may expose their unborn fetus to ethanol. The reversal of these effects by TUDCA, which inhibits ER and oxidative stresses, suggests that cellular stress underlies increased gluconeogenesis and glucose intolerance in rats prenatally exposed to ethanol, and that TUDCA has the potential to offset the hyperglycemia associated with prenatal ethanol.
